# Methylation Markers in Cutaneous Melanoma: Unravelling the Potential Utility of Their Tracking by Liquid Biopsy

**DOI:** 10.3390/cancers13246217

**Published:** 2021-12-10

**Authors:** Valentina Aleotti, Cristina Catoni, Cristina Poggiana, Antonio Rosato, Antonella Facchinetti, Maria Chiara Scaini

**Affiliations:** 1 Immunology and Molecular Oncology Unit, Veneto Institute of Oncology IOV-IRCCS, 35128 Padua, Italy; valentina.aleotti@iov.veneto.it (V.A.); cristina.catoni@iov.veneto.it (C.C.); antonella.facchinetti@unipd.it (A.F.); mariachiara.scaini@iov.veneto.it (M.C.S.); 2 Department of Surgery, Oncology and Gastroenterology, Oncology and Immunology Section, University of Padua, 35128 Padua, Italy

**Keywords:** melanoma, liquid biopsy, DNA methylation, biomarkers, circulating melanoma cells, cell-free circulating tumor DNA, tumor extracellular vesicles

## Abstract

**Simple Summary:**

Malignant melanoma is the most lethal form of skin cancer. While new therapeutic approaches have improved survival in patients with metastatic melanoma, responses are rarely sustained due to the high degree of heterogeneity at the inter- and intra-metastatic levels. The development of reliable biomarkers to monitor therapeutic response and disease progression is critical. While attention has been focused on dissecting the molecular basis responsible for treatment resistance, it is clear that epigenetic changes warrant further in-depth investigation. Indeed, many aberrantly methylated genes play a role in cell cycle control, apoptosis, and cell invasion, as well as in melanoma progression. Longitudinal monitoring of DNA methylation via liquid biopsy can provide real-time information on the behavior and stage of melanoma.

**Abstract:**

Malignant melanoma is the most serious, life-threatening form of all dermatologic diseases, with a poor prognosis in the presence of metastases and advanced disease. Despite recent advances in targeted therapy and immunotherapy, there is still a critical need for a better understanding of the fundamental mechanisms behind melanoma progression and resistance onset. Recent advances in genome-wide methylation methods have revealed that aberrant changes in the pattern of DNA methylation play an important role in many aspects of cancer progression, including cell proliferation and migration, evasion of cell death, invasion, and metastasization. The purpose of the current review was to gather evidence regarding the usefulness of DNA methylation tracking in liquid biopsy as a potential biomarker in melanoma. We investigated the key genes and signal transduction pathways that have been found to be altered epigenetically in melanoma. We then highlighted the circulating tumor components present in blood, including circulating melanoma cells (CMC), circulating tumor DNA (ctDNA), and tumor-derived extracellular vesicles (EVs), as a valuable source for identifying relevant aberrations in DNA methylation. Finally, we focused on DNA methylation signatures as a marker for tracking response to therapy and resistance, thus facilitating personalized medicine and decision-making in the treatment of melanoma patients.

## 1. Introduction

Malignant melanoma is the most lethal form of skin cancer, accounting for more than 59,000 deaths worldwide [1]. Indeed, although cutaneous melanoma is usually curable through surgical resection in patients with localized disease, the prognosis is less favorable in patients with regional metastases and advanced disease. Survival has historically been poor due to the infrequent response to conventional chemotherapy. However, in recent years, the systemic treatment of cutaneous melanoma has shifted dramatically as a result of the growing interest and focus on its genetic landscape. A combined approach, consisting of BRAF plus MEK inhibitors, has led to a significant improvement in overall survival (OS) for patients harboring a BRAF V600E/K mutation (35–50% of melanomas) [2,3,4]. Consequently, the definition of the tumor genetic landscape and the identification of molecular predictive factors have become critical for patients with unresectable disease and/or distant metastases [5]. Nonetheless, complete response to MEK/BRAF targeted therapy in approximately 50% of cases is only transitory, and great efforts have been made to characterize the mechanisms underlying the rise of resistance [6]. The primary impediment to achieving a durable response is the high degree of heterogeneity at the inter- and intra- metastatic levels. Therefore, it is now vital to develop reliable biomarkers to monitor therapy response and disease progression [7]. Apart from the effect given by their combined mechanisms of action, there is some evidence that BRAFi/MEKi treatment may promote a favorable immune microenvironment, thus enhancing the efficacy of immune checkpoint inhibition by anti-PD-1 drugs [8,9,10]. Nonetheless, even when used alone, immunotherapy with CTLA-4 and PD-1/PDL-1 blocking antibodies results in a durable response (approximately 2 years), but only in a subset of patients [11,12]. Although new therapeutic approaches have dramatically improved the survival of metastatic melanoma patients, the response is rarely sustained. Many efforts are nowadays concentrated on the dissection of the molecular basis putatively responsible for resistance to treatment, but the whole melanoma molecular landscape remains puzzling and warrants further investigation. Indeed, the extreme hypermutability and heterogeneity of the disease push toward a more complete and exhaustive description of the genomic landscape, able to define the dynamics of the systemic disease. Many studies using next-generation sequencing (NGS) have identified several genetic aberrations [13,14,15,16] that provide insights into the heterogeneity of melanoma, with putative implications for prognosis and therapy. NGS, indeed, can provide a comprehensive mutation profile, and thanks to the development and validation of numerous pipelines, it can successfully identify multiple target alterations with a reduced clinical reporting time [17,18]. Interestingly, the first comprehensive catalog of somatic mutations from an individual cancer, at the whole-genome level, concerned a melanoma cell line [19,20]. The catalog provided remarkable insights into the mutational signature, indicating the presence of a great number of mutations per Mb suggestive of DNA damage due to ultraviolet light exposure. Subsequent Whole Exome Sequencing (WES) studies, performed on clinical samples, demonstrated that *NF1*, *ARID2*, *PPP6C*, *RAC1*, *SNX31*, *TACC1*, and *STK19* were significantly mutated in melanoma [13,14]. Moreover, in the era of precision medicine and targeted therapies, molecular subtyping of melanoma has become more and more important by going complementary to the traditional clinicopathological classification [21]. Great support in this direction has been given by the exome sequencing studies of the Cancer Genome Atlas Skin Cutaneous Melanoma (SKCM-TCGA) project that classified cutaneous melanoma into four distinct molecular subtypes: BRAF-mutant, NRAS-mutant, NF1-mutant, and triple-wild-type group [15]. Moreover, through exome sequencing, previously reported melanoma onco- and tumor suppressor- genes have been confirmed (*BRAF*, *NRAS*, *CDKN2A*, *TP53*, and *PTEN*), and several additional mutated melanoma genes have been identified (*MAP2K1*, *IDH1*, *RB1*, and *DDX3X*). In the triple wild-type group, low-frequency mutations were also detected in *KIT*, *CTNNB1*, *GNA11*, and *GNAQ* [15,20]. A large, high-coverage Whole Genome Sequencing (WGS) study of cutaneous, acral, and mucosal melanoma was set a few years later by Hayward and colleagues [16]. This work supported the involvement of the non-coding genome in melanoma pathogenesis and disclosed different tumorigenic processes among the different melanoma subtypes [16,20]. Interestingly, the number of genes affected by recurrent non-coding mutations (predicted to have a functional impact) was equivalent to that of genes affected by recurrent coding mutations. Interestingly, eight genes, in addition to *TERT*, showed potential driver promoter mutations (*BLCAP*, *KBTBD8*, *NSUN6*, *RALY*, *RNF185*, *RPL29*, *RPS27*, and *ZNF778*). Nonetheless, beyond the genetic landscape depicted above, extensive research into epigenetic modifications is also required to find additional targets and treatment strategies [22,23]. Aberrant promoter methylation, for example, can affect gene expression; its hyper- and hypo- methylation is associated with gene silencing or overexpression, respectively. As already documented, many aberrantly methylated genes play a role in cell cycle control, apoptosis, cell invasion, and, ultimately, melanoma progression [24,25]. One of its strengths is that methylation is a promising biomarker due to the stability of methylated CpG islands (CGIs) in comparison to the hypervariability of melanoma mutational profiles [26].

## 2. DNA Methylation and Melanoma

### 2.1. DNA Methylation at a Glance

**Figure 1:**
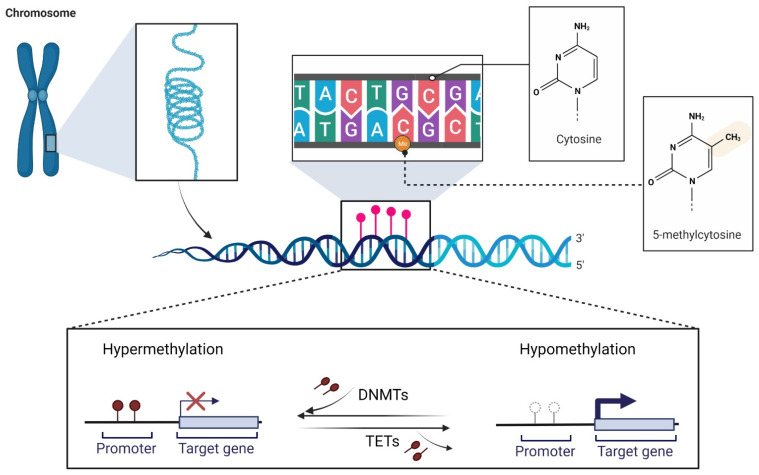
Schematic mechanism of DNA methylation and demethylation. In the DNA sequence, unmethylated cytosines are converted to 5’-methylcytosines (addition of the -CH3 group) by DNMTs. This event encompasses the CpG islands enriched in gene promoters and is generally associated with gene silencing. This reaction is potentially reversible due to the activity of TET enzymes, resulting in the loss of DNA methylation (hypomethylation). The white circles indicate unmethylated CpG sites, and the red circles denote methylated CpG sites. The crossed arrow on the left indicates the absence of transcription after DNA promoter methylation. The thick arrow on the right indicates the start of gene transcription as a consequence of promoter demethylation. Abbreviations: DNMTs, DNA methyltransferases; TETs, ten-eleven translocation methylcytosine dioxygenases. The above diagram was created using BioRender (https://biorender.com/ (accession date 8 October 2021)).

DNA methylation, along with histone modifications and non-coding RNAs (ncRNAs), is classified as an epigenetic modification. Epigenetic modifications are defined as heritable alterations in gene expression that occur without modifying the DNA sequence [27,28,29]. As is the case with several other physiological mechanisms, when improperly activated or silenced, it can affect the expression of oncogenes or tumor suppressor genes (TSGs), resulting in tumor initiation, development, and progression [30,31]. DNA methylation consists of the covalent addition of a methyl group (-CH3) to the fifth position of the pyrimidine ring of the cytosine nucleotide (5-methylcytosine, 5-mC) [28,29]. DNA methylation can putatively occur at any cytosine nucleotide in the genome, but its distribution is not random and is mostly restricted to the so-called CpG dinucleotides. CpGs are more abundant at gene promoters, where they tend to congregate and form CGIs [28,29,30]. Two main enzyme families are involved in a balanced and regulated manner in DNA methylation and demethylation processes [28]. DNA methyltransferases (DNMTs), which establish and maintain methylation patterns [30], and ten-eleven translocation (TET) methylcytosine dioxygenases, which are involved in the demethylation pathway (Figure 1) [32,33]. Under normal conditions, DNA methylation encompasses the entire genome, with the exception of short unmethylated regions within the CGIs [34]. The deregulation of DNA methylation mechanisms, characterized mainly by hypermethylation of CGIs in TSG promoters or global loss of DNA methylation, is common in cancer. CGI hypermethylation in promoter regions has been shown to affect genes involved in the regulatory circuits that control cell proliferation and homeostasis, enabling malignant cells to sustain their abnormal growth [29]. Cancer-associated promoter hypermethylation may affect between 5–10% of promoters containing CGIs [35]. Global loss of DNA methylation leads to molecular consequences that are advantageous to tumor development, including the generation of chromosomal instability [36,37], the loss of genomic imprinting [38], and the reactivation of transposable elements such as *LINE-1* [29,39,40].

### 2.2. DNA Methylation in Melanoma Development

**Figure 2:**
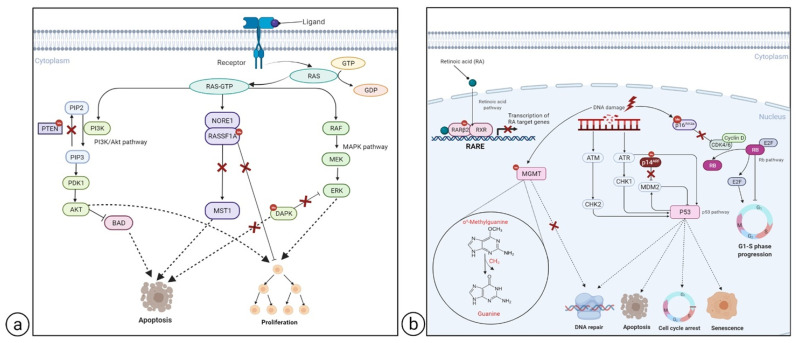
Pathways and genes involved in melanoma development. Aberrantly methylated genes and signal transduction pathways frequently altered during melanoma development and/or progression. (**a**) Shows the MAPK and PI3K-Akt pathways; while (**b**) depicts the p53, Rb, retinoic acid signaling, and DNA repair pathways. Hypermethylated TSGs are marked with a red dot; the alteration induced in the downstream pathway by TSG hypermethylation is represented by a red cross. The diagrams were created by means of https://biorender.com/ (accession date 8 October 2021).

Confined hypermethylation at CpG islands and global hypomethylation are epigenetic hallmarks of melanoma, both of which influence tumor behavior [41,42,43]. Melanoma initiation and progression have been associated with loss of tumor suppressors and oncogene activation [43]. Indeed, many TSGs are known to be aberrantly regulated via inactivation caused by specific methylation in the promoter region [44]. These genes appear to be involved in various signaling pathways, which are frequently altered during melanoma development and evolution. These pathways encompass the protein kinase activated by mitogen (MAPK), the phosphoinositide 3-kinase (PI3K), the tumor suppressor retinoblastoma (pRb), and the p53 protein pathways [41,43,45,46] (Figure 2). Since they can act synergistically or independently to control growth and apoptosis, their prolonged and uncontrolled activation is linked to proliferation, invasion, and metastasis. A gradual gain in DNA hypermethylation has been observed to increase in parallel with tumor aggressiveness and is known as the CpG island methylator phenotype (CIMP). It has been suggested that the genes involved in this increasing hypermethylation pattern form the melanoma CIMP [47]. Although less studied, DNA hypomethylation is equally important in the initiation and progression of melanoma. As previously stated, hypomethylation promotes tumor progression by causing genome instability via the demethylation of transposons and pericentromeric repeats, as well as the activation of specific oncogenes [43,48].

A detailed description of all the above aspects connected to melanoma driver epigenetic alterations is included in the next section.

#### 2.2.1. Hypermethylated Genes

As already stated, promoter hypermethylation of tumor-related genes is one of the major mechanisms for gene function loss. Moreover, there is a continuously updating number of tumor-related genes, including TSGs, that have been identified as being silenced as a result of the aberrant hypermethylation of CpG-rich promoter regions [49,50,51,52]. This type of TSG silencing mechanism is frequently found in multiple tumors and, together with the mutation-induced mechanism, is a potent means of disrupting TSG function. A large number of genes have been identified as hypermethylated in melanoma [41,53,54,55], including *O6-methylguanine-DNA methyltransferase* (*MGMT*) [52,56], cyclin-dependent kinase inhibitor 2A (*CDKN2A*) [52,57], and Ras-association domain family 1 isoform A (*RASSF1A*) [49,58] (Table 1). Interestingly, some of them have been found to contribute to melanoma progression and have been associated with aggressive clinicopathological features and poorer survival [53,55]. Some of the most frequent and best characterized hypermethylated genes are described below. The pathways in which they act are highlighted in Figure 2.

##### *RASSF1A* 

The Ras-association domain family 1 (*RASSF1*) gene, located in the 3p21.3 region, contains eight exons and gives rise to eight different transcripts, from *RASSF1A* to RASSF1H, by means of alternative splicing and two different promoters [98]. The *RASSF1A* tumor suppressor is a scaffold protein involved in cell signaling. This protein is situated at the intersection of a complex signaling network that includes key regulators of cellular homeostasis such as RAS, MST2/Hippo, p53, and death receptor pathways [56]. Notably, *RASSF1A* is involved in the inhibition of cell proliferation and, because its signaling is regulated by pro-apoptotic insults, it activates intrinsic and extrinsic apoptotic cascades [99,100]. The hypermethylation of its promoter and subsequent gene downregulation has been observed in melanoma cell lines, tissues, and serum of stage III/IV cutaneous melanoma patients [47,49,55,65,76,78,93,95,96], and a significant proportion of uveal melanoma [77,97]. Several methods have been used to assess the methylation status of *RASSF1A* promoter, including methylation-specific polymerase chain reaction (MSP) [47,49,65,76,78,93], real-time quantitative MSP [76], methylation-specific multiplex ligation-dependent probe amplification (MS-MLPA) [55], real-time qPCR [95], and semi-nested PCR [96]. Inactivation of *RASSF1A* may play an important role in the selective advantage of melanoma cells. In particular, the high frequency with which *RASSF1A* is inactivated suggests that this could be an important alternate pathway for disrupting Ras signaling [49].

##### *DAPK* 

The death-associated protein kinase (*DAPK*), located on 9q34.1, encodes a serine/threonine kinase belonging to the calmodulin-regulated kinase (CAMK) superfamily, which also includes DRP-1 and ZIP-kinase (ZIPK) [101]. It plays a key role in tumorigenesis by acting as a regulator of caspase-dependent and caspase-independent cell death. *DAPK* is necessary for apoptosis induced by multiple death signals [102,103] and has also been associated with autophagy activation [103,104,105]. It functions as a tumor suppressor, and its loss occurs as a result of promoter methylation in many cancers [106,107]. *DAPK* hypermethylation has been detected through MSP investigation in 19% of metastatic tumor tissues from stage III/IV melanoma patients [76] and in only 5% of uveal melanoma tissues (real-time quantitative MSP analysis) [77]. The epigenetic silencing of *DAPK*, along with *RARβ2*, *RASSF1A*, and *MGMT*, has been observed as a common mechanism of tumor formation in cutaneous melanoma [76]. This may be due to *DAPK*’s inability to suppress cellular transformation during the early stages of tumor development [108] and to inhibit metastasis [101,109,110].

##### *PTEN* 

The phosphatase and tensin homolog gene (*PTEN*) is located on 10q23.31 and encodes a phosphatase that acts as a negative regulator of the PI3K/Akt pathway by dephosphorylating phosphatidylinositol (3,4,5)-triphosphate (PIP3), an important intracellular second messenger necessary for the activation of the Akt protein, a serine/threonine kinase involved in cell growth and survival [89,111]. The loss of functional *PTEN* leads to the downregulation of apoptosis and/or increased proliferation [111]. In melanoma, *PTEN* hypermethylation and constitutive activation of the Akt pathway promotes tumor progression. Indeed, *PTEN* has been shown to be frequently hypermethylated in malignant melanoma, and a subsequent reduction in its expression in both tissue and serum samples has been noted [55,90,91]. Although the biological impact of the hypermethylated *PTEN* promoter in melanoma is still not fully elucidated, several studies have examined its prognostic significance [92], and the association of *PTEN* methylation with advanced stage has been demonstrated. Indeed, an increase in its methylation, detected by MS-MLPA, has been observed in stage III/IV patients compared to early-stage primary tumors, resulting in reduced OS and disease-free survival (DFS) [55].

##### *CDKN2A* 

The cyclin-dependent kinase inhibitor 2A (*CDKN2A*) gene, located on 9p21.3, encodes two tumor suppressor proteins that are frequently mutated in human cancer [52], including melanoma [66]. These two proteins, *p16^INK4A^* and *p14^ARF^*, are important regulators of the retinoblastoma (Rb) and p53 pathways, respectively, and their gene promoters have been found to be hypermethylated and, consequently, silenced in both primary and metastatic melanoma [65,67].

At the G1-to-S transition in the cell cycle, *p16^INK4A^* specifically inhibits the cyclin D-dependent kinase 4/6 (CDK4 and CDK6)-mediated phosphorylation of the retinoblastoma protein (pRB), thus sequestering E2F transcription factors and consequently blocking cell cycle progression [68,69,70,71,112]. Whereas the expression of the *p16^INK4A^* protein leads to cell cycle arrest, the *p14^ARF^* protein mediates the degradation of MDM-2, which increases p53 protein levels, thus inducing cell cycle arrest to allow for either DNA repair and cell survival or apoptosis to discard the damaged cell [68,69,112]. The *p16^INK4A^* promoter is found to be hypermethylated and, consequently, silenced in several cancer types [113]. While the hypermethylated *p16^INK4A^* promoter, identified by MSP, was found in 19% of primary cutaneous melanoma cases, the frequency tends to be higher in metastases (33%), indicating that hypermethylation may also occur in later stages [65]. It is associated with increased tumor cell proliferation and reduced patient survival. It is worth noting that *p16^INK4A^* promoter methylation, analyzed by MSP, has been predominantly observed in 52% of NRAS-mutated metastatic melanomas but in only 7% of BRAF-mutated metastases [66]. *p14^ARF^* has been shown to be hypermethylated in approximately 57% of metastatic melanoma tumors, independently of the *p16^INK4A^* promoter [67].

##### *MGMT* 

The *O6-methylguanine DNA methyltransferase* (*MGMT*) gene is located on 10q26.3 and encodes an enzyme involved in repairing damaged guanine nucleosides [114]. *MGMT* plays a central role in preventing normal cells from transforming into tumor cells and also protects tumor cells from the cytotoxic effects of chemotherapy with alkylating agents, such as temozolomide (TMZ) and dacarbazine (DTIC), widely used in treating melanoma and glioblastoma [82,115]. This protein catalyzes the transfer of methyl groups from the *O6* site of guanine in DNA to its cysteine residue (the protein active site) [116]. *MGMT* is silenced during the oncogenesis of several human cancer types, leading to inefficient repair of DNA alkylation, and resulting in increased sensitivity to alkylating agents and decreased tumor survival [117]. The hypermethylated state of *MGMT* has been detected in the cell lines, tissues, and serum of melanoma patients using MSP [65,72,76,78,83], real-time quantitative MSP [76], high-resolution melting point analyses (HRMA), pyrosequencing [83], and MS-MLPA [84]. It is worth noting that the epigenetic silencing of *MGMT* has been associated with a significantly improved response to DTIC/TMZ single-agent therapy, a longer progression-free survival (PFS) in stage IV melanoma patients [83], and prolonged survival in stage III melanoma patients locoregionally treated with melphalan [84]. All this evidence suggests that the *MGMT* methylation status may be used as a predictive biomarker of favorable survival in patients receiving chemotherapy [83,84].

##### *RARβ2* 

The *Retinoic Acid Receptor Beta 2* (*RARβ2*), located on 3p24.2, encodes a retinoid-inducible tumor suppressor protein. It is a member of the nuclear receptor superfamily, which also includes RARα and RARγ, both of which are expressed differentially during development and adulthood. Together with retinoid X receptors (RXRs), RARs are bound and activated by the biologically active forms of vitamin A (all-trans retinoic, ATRA; 9-cis-retinoic acid, CRA). The activated heterodimer of RAR and RXR binds to the retinoic acid receptor responsive element (RARE) and regulates the transcription of the target genes involved in cell differentiation, proliferation, and apoptosis [118]. Several approaches, including MS-MLPA [55], MSP [76,78,93], real-time quantitative MSP [76,94], and pyrosequencing [85], have been used to disclose that the promoter of this gene is frequently hypermethylated in the cell lines, tissues, and serum of melanoma patients [55,76,78,85,93,94].

In addition to the other TSGs found to be frequently hypermethylated in melanoma, Hoon and colleagues reported that the hypermethylation of *RARβ2* was significantly correlated with Breslow thickness, which is an important prognostic factor in patients with early-stage melanoma [76]. The authors concluded that the silencing of this gene might be a key epigenetic factor in melanocyte transformation and primary lesion progression. These results were also confirmed in a more recent study, where a significant reduction in DFS and OS was found in the presence of *RARβ2* hypermethylation [55].

##### CpG Methylator Phenotype (CIMP) in Melanoma

The CpG methylator phenotype (CIMP) was first observed in colorectal cancer [119] and consists of a gradual increase in the level of CGI methylation along with tumor aggressiveness. This phenomenon has also been observed by Tanemura and colleagues in melanoma patients [47]. In this work, they investigated the significance of the CGI methylation status in the progression of malignant melanoma by means of MSP and absolute quantitative assessment of methylated alleles (AQAMA). Methylation of several tumor-related genes, including *MINT17*, *MINT31*, *TFPI2*, *WIF1*, *RASSF1A*, and *SOCS1*, comprising the first melanoma CIMP, increased significantly with advanced clinical stages, suggesting that their inactivation is associated with tumor progression. In recent years, a DNA methylome profiling study, performed by the Illumina Infinium HumanMethylation450 BeadChip, detected additional hypermethylated genes involved in the invasiveness of primary and metastatic melanomas, including *ARHGAP22* and *NAV2* [53]. In addition, it was recently observed that melanoma CIMP associated with an NRAS-mutant phenotype is more aggressive than melanoma CIMP associated with BRAF-mutant melanoma [120]. However, the strong association between BRAF V600E and CIMP that emerges in colorectal cancer is not observed in melanoma [121]. Although the underlying mechanism of CIMP is still unclear, these data suggest a tissue-specific biological mechanism rather than a universal one for all cancers [121]. All these findings could be essential for developing novel tools to identify early metastasis-promoting epigenetic events.

#### 2.2.2. Hypomethylated Genes

It has been observed that malignant tumor tissues are characterized by global DNA hypomethylation when compared to normal tissues or benign tumors [122,123]. To quantify the global methylation level of an entire genome, it is common practice to analyze the methylation level of the Long Interspersed Nuclear Element-1 (*LINE-1*) sequences (approximately 20% of the human genome [124]), representing a surrogate marker for global methylation status [125,126]. Global demethylation has been identified as a putative hallmark of the metastatic capacity of primary melanomas [127]. Based on previous studies demonstrating a correlation between *LINE-1* hypomethylation and CGI hypermethylation of TSGs [128,129], Hoshimoto and colleagues evaluated the methylation status of *LINE-1* and the TSG AIM1 in melanoma patients in order to develop a combination of biomarkers with prognostic utility. Tissue analysis by AQAMA revealed that *LINE-1* hypomethylation was higher in stage IV melanoma than in other stages and that AIM1 hypermethylation (MSP detection) was more frequent in metastatic melanoma than in primary melanoma (65% vs. 38%). The combination of *LINE-1* hypomethylation and AIM1 hypermethylation was a significant predictor of DFS and OS in stage I/II patients. In addition, they observed that patients with *LINE-1* hypomethylation or AIM1 hypermethylation in serum had a worse prognosis [130]. Apart from the global hypomethylated pattern observed in melanoma, specific hypomethylated genes have been identified and correlated with patient outcome (Table 2).

##### *TBC1D16* 

It has been observed that the hypomethylation of the *TBC1D16* gene, which encodes, among others, the TCB1D16-47KD isoform, generates metastases in melanoma [138]. More in detail, Vizoso and colleagues exploited the use of the Illumina Infinium HumanMethylation450 BeadChip for screening genes with differential DNA methylation in both primary tumor-derived and metastatic melanoma cell lines. This approach led to the identification of the TCB1D16-47KD isoform, whose gene promoter was hypermethylated and downregulated in primary tumor–derived cell lines but was not unmethylated and upregulated in metastatic cell lines. Finally, it was observed that *TBC1D16-47KD* hypomethylation in metastatic patients was associated with a shorter PFS and OS. Worth noting is that this unmethylated state was observed in 33% of patients carrying the BRAF V600E mutation and has been associated with increased sensitivity to BRAF inhibitors [138].

##### *PDGFD*, *ZEB1*, and *THRB*

There is currently no targeted therapeutic approach for NRAS-mutant melanomas. Starting from this consideration, Jiang and colleagues performed an integrative analysis encompassing DNA methylation, gene expression, and microRNA expression to identify downstream pathways affected by the most common NRAS driver mutations (Q61K/L/R). DNA methylation profiles of 61 primary melanomas, processed through the Illumina Infinium HumanMethylation450 BeadChip, and obtained from TCGA, were examined [136]. First, global hypomethylation induced by or associated with the NRAS^Q61^ driver mutation was identified as a common feature in melanoma (similar to what Hou and colleagues demonstrated for BRAF V600E signaling), potentially playing an important role in the disease pathogenesis [24,136]. NRAS^Q61^ mutations were then linked to the hypomethylation of *PDGFD*, *ZEB1*, and *THRB* genes, resulting in their increased expression and the dysregulation of the involved downstream pathways. Finally, the expression of *PDGFD*, *ZEB1*, and *THRB* was also associated with patient survival time, thus suggesting that they may have therapeutic potential. *PDGFD* is located upstream of the MAPK and PI3K pathways and plays a role in the regulation of cell proliferation, transformation, invasion, and angiogenesis [140]. As a transcription factor involved in the epithelial to mesenchymal transition, *ZEB1* is an oncogene that can promote neoplastic transformation [141]. Finally, *THRB* is one of the thyroid hormone receptors [142], and defects in its gene are known to confer generalized thyroid hormone resistance, thus opening up new perspectives for the putative use of thyroid hormone therapy to treat NRAS-mutant melanomas [136].

##### *TKTL1* 

The reprogramming of energy metabolism to fuel uncontrolled cell growth and division is a hallmark of cancer. Certain tumor cells are part of a subpopulation of glucose-dependent cells (“Warburg effect”’) that secrete lactate, which is imported and preferentially used as fuel by other tumor cells, resulting in perfect cooperation [143]. Hypomethylation of Transketolase-like 1 (*TKTL1*), evaluated by quantitative methylation-specific PCR (MS-qPCR), appears to be involved in enhancing the Warburg effect in melanoma by accelerating glucose utilization and lactate production, thereby increasing the likelihood of the successful invasion of melanoma cells [139].

## 3. DNA Methylation in Melanoma Liquid Biopsies

**Figure 3:**
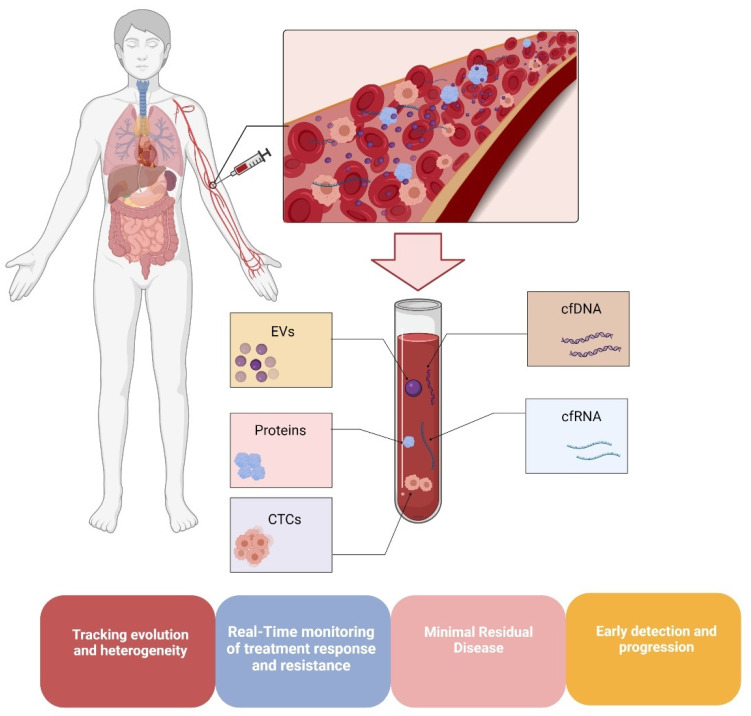
Liquid biopsy in cancer patients. Circulating tumor cells (CTCs), circulating cell-free tumor DNA (ctDNA), circulating cell-free RNA (cfRNA), and extracellular vesicles (EVs) can be isolated simultaneously from the same blood sample. Their analysis provides real-time information on tumor progression, minimal residual disease, treatment response, and resistance. Abbreviations: CTCs, circulating tumor cells; ctDNA, circulating cell-free tumor DNA; cfRNA, cell-free circulating RNA; EVs, extracellular vesicles. Diagram created by means of Biorender (https://biorender.com/ (accession date 8 October 2021)).

In this era of “personalized medicine,” much attention is being paid to different approaches that provide repeatable and safer insights into tumor evolution and heterogeneity. Since cutaneous melanoma is characterized by extreme heterogeneity and high tumor mutation burden (TMB) [144], the early detection of tumor-related changes such as chromosomal rearrangements, copy number variation, or mutations in oncogenes and tumor suppressor genes, is mandatory for the choice and adjustment of targeted therapy, treatment monitoring, and detection resistance [145]. In the last decade, the so-called “liquid biopsy” [146], which refers to a test of body fluids (i.e., blood, urine, and saliva), has emerged as a novel biomarker with significant application in translational research due to its ability to provide comparable (or more detailed) information than the conventional tissue biopsy [145]. Indeed, the analysis of circulating tumor cells (CTCs), cell-free circulating nucleic acids (cfDNA and cfRNA), and extracellular vesicles (EVs) via a minimally invasive blood draw has opened new avenues for cancer diagnostics, enhancing risk assessment, real-time monitoring of therapeutic efficacy, early detection of relapse, and monitoring of tumor evolution (Figure 3) [145,147,148,149]. Like all circulating tumor cells, Circulating Melanoma Cells (CMCs) are released into the bloodstream by the primary tumor and/or metastases [145,150]. Due to their high heterogeneity and rarity in the bloodstreams of metastatic melanoma patients [151,152], the continuous improvement of even more sensitive methods to detect and characterize them is of paramount importance. On the other hand, cell-free circulating DNA is produced from physiological functions such as apoptosis, necrosis, or secretion. Moreover, cancer patients have higher amounts of cfDNA than healthy controls [149,153]. Thus, the fraction of cfDNA that originates from tumor cells, called circulating tumor DNA (ctDNA), has been extensively studied as a putative disease marker, both in terms of quantity and composition. Indeed, the identification and monitoring of ctDNA using mutation-detection techniques (i.e., droplet digital PCR (ddPCR) and targeted next-generation sequencing (NGS) panels) has been well documented, and its potential as a promising biomarker for diagnosis, evaluation of treatment effectiveness, and as a tracker of tumor evolution has been well assessed in many cancers, including melanoma [149,154,155,156,157,158,159]. Finally, EVs actively participate in intercellular communication by taking part in the transfer of lipids, proteins, and RNA, thus suggesting a putative active role in cancer development [160,161]. Moreover, they have enormous potential as biomarkers, given that their tumor-derived cargo may be used for different applications, i.e., tumor burden estimation and survival prediction [145,162]. All things considered, the search for reliable biomarkers capable of tracking the disease evolution and heterogeneity in real-time may be successful in liquid biopsy, which is the sum of systemic disease. Since the use of targeted therapies and immunotherapy has significantly altered the natural history of melanoma, it is critical to closely monitor its genetic landscape to ensure its success. As previously stated, aberrant methylation of gene promoters can be a characteristic of cancer [163], and, as a result, the analysis of methylated DNA in liquid biopsy is an emerging field of interest [81,164,165]. It has already been demonstrated that hypermethylation of the promoters of certain selected genes can be used to distinguish melanoma patients from healthy individuals [81], demonstrating its utility as a diagnostic marker. Notably, DNA methylation patterns can change during melanoma progression; thus, longitudinal monitoring of DNA methylation in a non-invasive manner via liquid biopsy can provide real-time information about the behavior and stage of melanoma [80].

### 3.1. DNA Methylation in Circulating Melanoma Cells

To date, the CTC count as a prognostic/predictive marker is well established for several malignancies for which the cut-off has already been validated [166,167,168]. For others, melanoma included, the process is still ongoing. In any case, this biomarker has garnered considerable attention due to the type of information that its genetic characterization could offer, and more recently, the DNA methylation profile has attracted significantly more attention. However, the study of CTC methylome remains largely unexplored due to the lack of adequate investigation techniques. One of the first studies examined the simultaneous detection of CMCs, inferred by the identification of specific mRNAs in the blood and specific methylated genes. More in detail, Koyanagi and colleagues identified a correlation between the number of melanoma markers (MART-1, GalNAc-T, and MAGE-A3 mRNAs), detected in the blood through quantitative real-time reverse transcription-PCR assay, the presence of circulating methylated *RASSF1A* and *RAR-β2* genes (MSP detection), and patient outcome. Patients with both CMCs and methylated genes showed a significantly poorer response to biochemotherapy, as well as a shorter PFS and OS [169]. Salvianti and colleagues conducted a similar study, focusing on methylated cfDNA and CMCs as two complementary liquid biopsy biomarkers that can be combined to enhance the possibility of disease monitoring [95]. Indeed, they compared *RASSF1A* promoter methylation tracked in cfDNA by real-time qPCR with the presence of CMCs in both healthy controls and patients at different melanoma stages (in situ, invasive, and metastatic). They found that the percentage of cases with methylated *RASSF1A* promoter was higher in melanoma patients than in healthy subjects (46% vs. 10%), thus indicating *RASSF1A* promoter methylation as a good predictor of disease (AUC of 0.905). However, when they checked the presence of CMCs in the three different patient categories, they found no significant association with methylated *RASSF1A* tracked in cfDNA [95].

Currently, no studies have been conducted to determine the methylation status of CMCs. However, the few studies that have investigated CTC methylation in other cancers are very interesting. Recent publications have focused on finding the optimal technique for studying CTC methylome in lung [170], breast [171,172], and colon cancer [171]. More in detail, Zhao and colleagues developed an approach called LCM-µWGBS that combines laser capture microdissection (LCM) CTC capture and whole-genome bisulfite sequencing (µWGBS), enabling the analysis of a small number of CTCs to obtain information on their DNA methylation landscape [170]. They defined the DNA methylome of CTCs from lung cancer patients and compared it to the global DNA methylation of normal tissues. Interestingly, they found a progressive decrease in global DNA methylation from normal tissue to primary tumors and CTCs, suggesting a gradual loss of DNA methylation during tumorigenesis. Moreover, they observed a tendency toward the increased methylation of several TSG promoters in CTC-DNA compared to the primary tumor.

In a very recent study, Chen and colleagues, by means of single-cell bisulfite sequencing (scBS-seq), provided an important analysis of the single-cell DNA methylome in CTCs, characterizing tumor heterogeneity and the evolution of the tumor cell methylome during cancer progression [171]. Seventeen cancer patients covering six different types of cancers (lung adenocarcinoma, small-cell lung cancer, breast, colon, gastric, and prostate cancer) were tested to assess the methylation level in single CTCs [171]. Firstly, they observed that CTCs, similar to primary tumors, exhibit lower methylation levels than those in normal cells. They also observed that these cells show inter- and intra-patient heterogeneity in terms of promoter methylation. Interestingly, they also investigated the dynamic methylome changes, which occur during cancer metastasis, in the promoter regions of 20 known tumor-associated genes of primary, metastatic tissues, CTCs, and white blood cells of gastric cancer patients accompanied by abdominal ovarian metastasis. For instance, they observed that the methylation level of the *LTF* gene, often reported down-regulated in tumors, increased with cancer progression. The methylation level of several genes expressed in the ovaries, such as *1orf35*, *DENND6A*, and *ZNF285*, decreased transiently. Furthermore, the methylation level of genes involved in oncogenic transformation- and cell adhesion-associated pathways (*FBP2*, *HIVEP3*, *PTPN21*, and *CEACAM5* genes) decreased, suggesting a role for these pathways in tumor progression.

Since CTCs can be found in patient blood as single CTCs or CTC clusters, Gkountela and colleagues found that CTC clusters are distinguishable from single CTCs based on their methylation status, assessed by single-cell whole-genome bisulfite sequencing. They observed that in CTC clusters, hypomethylated regions are associated with key regulators of stemness and metastasis (*OCT4*, *NANOG*, *SOX2*, and *SIN3A*), while hypomethylated regions in single CTCs are independent of the pluripotency network [172].

### 3.2. Methylation in Circulating Melanoma DNA

Several recent studies have focused on ctDNA methylation screening and its potential clinical application, in addition to genomic and/or expression analyses [173,174]. Furthermore, most recent studies have focused on the analysis of ctDNA rather than FFPE samples in an attempt to overcome the limitations associated with the poor quality of the FFPE genetic material. In one of the first studies, based on real-time MSP detection, Hoon and colleagues examined the presence of hypermethylated TSG promoters in the serum of melanoma patients [76]. They observed that the incidence of TSG hypermethylation increased during tumor progression and that *MGMT*, *RASSF1A*, and *DAPK* hypermethylation were significantly lower in primary melanomas compared to metastatic ones. On the other hand, the frequency of hypermethylated RAR-ẞ2 was similar in both primary and metastatic melanomas [76].

A subsequent study by Mori and colleagues highlighted the utility of detecting circulating methylated tumor-related genes in serum (MSP analysis) as a predictive marker of response to biochemotherapy and OS. Among the most frequently hypermethylated genes in melanoma, they discovered a significant correlation between the hypermethylation of *RASSF1A* and RAR-ẞ2, the response to biochemotherapy, and OS [78]. Interestingly, this correlation with treatment response was not observed when analyzing the *MGMT* methylation status [78].

A recent study by Liu and colleagues focused on assessing the feasibility of using cfDNA methylation profiles in advanced cancer patients for the detection of metastatic disease [173]. They developed an NGS targeted methylation sequencing assay to measure the methylation status of more than 9000 CpG sites, selected according to TCGA data, and parallel classify the presence of advanced cancer, being also able to predict tumor origin (i.e., melanoma, colorectal cancer, NSCLC, and breast cancer). The authors demonstrated that plasma cfDNA methylation scores detected the presence of cancer in 83.8% of cancer patients with 100% specificity and predicted cancer type in 78.9% of cases. Focusing on melanoma, Diefenbach and colleagues developed an efficient ctDNA methylation analysis workflow using an amplicon-based NGS panel performed on bisulfite-treated DNA. They confirmed the hypermethylation of seven genes (*GJB2*, *HOXA9*, *MEOX2*, *OLIG3*, *PON3*, *RASSF1*, and *TFAP2B*), known to be hypermethylated in metastatic melanoma patients but not in healthy individuals [81]. Moreover, in contrast to previous studies on cfDNA methylation, they were able to examine the methylation of symmetrical CpG sites in both DNA strands to accurately quantify the level of gene methylation [81].

Some studies have explored the potential of methylation pattern tracking in early-stage tumors. In this regard, the clinical validity of a targeted methylation-based Multi-Cancer Early Detection (MCED) test using cfDNA sequencing has been investigated in several cancer types, including melanoma [174]. This new approach aims to provide the tools to detect tumors at an earlier stage, thus reducing cancer mortality by identifying the cancer signal origin (CSO) [174,175]. The MCED test demonstrated a specificity of 99.5% (false-positive rate of 0.5%) and an overall sensitivity of 51.5% for cancer signal detection. The authors suggested to use this test either in combination with other single-cancer screening tests (for the detection of breast, colorectal, cervical, lung, and prostate cancers) or as a screening for those cancers for which tests are not yet available in the United States. However, the high costs of this type of analysis could represent an obstacle for its application in the clinical setting [174].

In summary, the role of DNA methylation in melanoma deserves deeper investigation, as its role in tumorigenesis is only partially understood. Its usefulness as a biomarker could be helpful in defining a precision medicine workflow. In particular, a suitable and optimal analysis technique should be sought, which is currently lacking. In addition, a cost-benefit analysis should be conducted. While whole-genome bisulfite sequencing can provide a complete methylation profile at a very high cost, a PCR-based approach is cheaper but assesses only a small number of CpG sites [173]. The use of NGS panels, which allows simultaneous analysis of the methylation status of several tens to hundreds of genes, could be an alternative and, if customized and restricted to a limited number of regions of interest, might be an excellent compromise to limit costs.

### 3.3. Methylation in Melanoma Extracellular Vesicle-Derived DNA (evDNA)

In recent years, there has been growing interest in understanding the role of extracellular vesicles (EVs) in melanoma progression since they are considered to be an alternative means of intercellular communication. EVs are composed of a heterogeneous group of membrane-delimited nanoparticles, usually classified into exosomes (30–120 nm), microvesicles (100–1000 nm), and apoptotic bodies (500–4000 nm) based on their size [176]. They play a key role in the delivery of active cargoes, including DNA fragments, coding and non-coding RNA, proteins, and lipids, from donor to distal cells, and they are released by normal and cancerous cells into the external microenvironment [177]. Tumor-derived EVs have been shown to affect the pathophysiology of recipient cells, modulating a variety of processes involved in cancer progression (increased invasiveness, proliferation rate, and chemoresistance) [178].

The study of EV content is emerging as an innovative and helpful strategy to understand tumor processes due to the fact that the bioactive molecules are packaged and protected within the phospholipid bilayer of EVs. Although research on EV-derived RNA and proteins has been largely explored, only a few studies have been conducted on EV-associated DNA (evDNA). Recent evidence suggests that most of the DNA associated with tumor-derived EVs is double-stranded (dsDNA), representing the entire genome and informing on the mutational status of parental tumor cells [179]. However, the mechanism of DNA loading onto EVs remains unclear. The main hypothesis is the encapsulation of cytosolic DNA during EV biogenesis [160]. In recent years, the methylation analysis of evDNA has attracted attention as a biomarker for the detection of various cancers. The benefit of using evDNA is its stability and protection from digestive enzymes due to encapsulation in the lipid bilayer of EVs. However, major steps forward are needed to overcome issues related to the source of evDNA, sample collection, and DNA extraction methods that appear to affect methylation detection. For example, it is necessary to develop a better strategy for isolating evDNA without cfDNA contamination because it has been observed that DNA tends to stick to any surface, including the lipid envelope of EVs, resulting in the possible co-isolation of cfDNA with the EV during the purification protocol [180]. In this regard, the digestion of the EV pellet by DNAses may be an optimal strategy to overcome the co-isolation of nucleic acids with EVs. The DNA extraction efficiency largely depends on which method of EV isolation and evDNA extraction is used. Moreover, the presence of DNA in exosomes continues to be controversial. In 2019, while Coffey and colleagues concluded that exosomes do not contain DNA, Yokoi and colleagues observed genomic DNA and nucleoprotein in them. Perhaps it may depend on an over-strict exosome isolation strategy that can lead to the loss of evDNA, the levels of which are too low to be detected. Because the most effective approach for EV isolation has not yet been well established, and different methods of EV isolation and DNA extraction are currently used, it is mandatory to develop an optimal common strategy for downstream applications. For example, García-Romero and colleagues compared the most common EV-isolation methods and found that polyethylene glycol precipitation (PEG) seems to be the most feasible and affordable EV isolation technique [181]. Moreover, in a study by Kamyabi and colleagues, a microfluidic platform was described as a method for the rapid isolation of EVs from the plasma of pancreatic cancer patients [182].

Although data on melanoma are still limited, and most of the data are restricted to in vitro experiments, overall findings suggest that the methylation profile of evDNA shows similarities in methylation profile with that of genomic DNA (gDNA) in murine melanoma cells [179]. This evidence has also been confirmed in other cancer types, such as metastatic castration-resistant prostate cancer [183], diffuse large B-cell lymphoma [184], and glioblastoma [185]. A new and interesting approach was recently developed using an electrochemical detection method based on the differential absorption capacities of different methylation levels of DNA on a gold surface [177]. Using this highly sensitive microdevice, Sina and colleagues performed exploratory research to develop a method to isolate evDNA from ctDNA based on methylation-dependent physicochemical properties. They found that evDNA has surface-based properties similar to cellular gDNA, but not to cfDNA, probably due to the longer size of gDNA. Moreover, they demonstrated that this method was able to discriminate cancer and normal evDNA, suggesting a potential use of this device for clinical applications (the adsorption level on the gold surface of patient evDNAs was 20–40%, whereas that of the normal evDNAs was below 20%) [177].

In addition, the promoter regions of specific genes in metastatic castration-resistant prostate cancer (GSTP1, *RASSF1A*, and *SLFN11*) and in diffuse large B-cell lymphoma (*CDKN2A* and *CDKN2B*) are found to be methylated in both evDNA and primary tumor tissue or CTCs, respectively [183,184]. These findings not only highlight the potential use of evDNA methylation analysis as a biomarker for the detection of cancer but also point toward a better understanding of the evDNA methylation profile in melanoma cancer, which is poorly understood.

## 4. Circulating Methylated DNA Biomarkers for Tracking Response to Therapy and Resistance Onset

The development of treatment resistance is the main limitation to many anti-cancer therapeutic approaches. DNA methylation has the potential to be used as a biomarker in many clinical situations, including for the prediction of response to therapies and the monitoring of recurrence [41]. As previously discussed, positive signals for a putative prognostic role for methylation status, tracked through liquid biopsy, were found in the early 2000s by Koyanagi and colleagues, who hypothesized the clinical utility of two different molecular variables (CMC-mRNA and serum methylated DNA) in a cohort of patients undergoing biochemotherapy [169]. Similarly, Mori and colleagues assessed the prognostic and predictive significance of detecting specific methylated genes in the serum of melanoma patients undergoing biochemotherapy [78]: they found that hypermethylation of *RASSF1A* was the best predictor of response and OS, thus corroborating previous evidence found in other malignancies of resistance to cisplatin and tamoxifen associated with *RASSF1A* hypermethylation [186,187]. Moreover, the methylated status of *RAR-β2* was significantly related to survival. As *RAR-β2* is involved in the control of cell growth and apoptosis, its expression in tumor cells susceptible to its mediated apoptosis could be an important biomarker of response to biochemotherapy [78]. A recent study by de Vos and colleagues involved a large patient cohort with different malignancies from the Cancer Genome Atlas (TCGA) Research Network to test the value of two hypermethylated genes, *SHOX2*, and *SEPT9*, as pan-cancer biomarkers [188]. The quantitative methylation analysis of these genes has shown a correlation with treatment response: when compared to conventional monitoring, cfDNA longitudinal screening revealed an association between an increase in the methylation score (CMS, cumulative cfDNA methylation score) and non-responsiveness to treatment with an 80-day advantage.

Nowadays, chemotherapy with two alkylating agents, DTIC or TMZ, is still an alternative in the event of the development of resistance to immune checkpoint inhibitors (ICI) and/or targeted therapy, or in the case of mutation-negative melanomas [43,189,190]. Therefore, biomarkers capable of identifying the minority of patients who may benefit from this type of approach would be critical, as they are currently lacking. The effects of methylating agents have been putatively related to the expression of *MGMT*. Its overexpression protects against cell death induced by alkylation. On the other hand, low expression is correlated with a higher possibility of responding to methylating agents [191,192]. As melanomas are likely to express low levels of *MGMT* [193,194], this could explain why they respond to methylating drugs, including DTIC and TMZ, but not to other anti-cancer drugs. The *MGMT* methylation status can thus determine the outcome in melanoma patients treated with methylating drugs. In fact, *MGMT* promoter methylation has been associated with response to single-agent DTIC/TMZ and longer PFS in disseminated cutaneous melanoma [83], even if conflicting data have been reported [190,195]. Melanoma cells appear to be intrinsically resistant to drugs and/or acquire resistance through different strategies involving multiple other players [195]. Ultimately, the simultaneous tracking of the *MGMT* methylation status and other players could be of great interest for determining the success of TMZ therapy, and liquid biopsy, as a sum of systemic disease, may be the optimal source for performing this type of longitudinal screening.

## 5. Methylation Markers from Bench to Bedside

Liquid biopsy evaluation of epigenetic biomarkers is an emerging field in oncology that might have a putative impact in improving diagnostic and screening procedures [196]. Several benefits may address the use of DNA methylation as a biomarker, such as its high stability in several biofluids and its dynamism during disease evolution. Speaking about the clinical utility of DNA methylation in cutaneous melanoma, some attempts have already been made in this direction: i.e., the *RASSF1A* promoter hypermethylation tracked in the cfDNA has been found to be a good diagnostic and prognostic biomarker [78,95]. However, several hindrances make the incorporation of this putative biomarker in the clinical routine still uncertain [197]: first, much efforts are currently being made by the scientific community to standardize the preclinical factors governing the handling of the liquid biopsy samples [198]. The choice of blood collection tubes, for example, is a critical pre-analytical variable [198] as in the clinical setting the fast processing is not always possible. Thus, the levels of the contamination of cfDNA derived from nucleated blood cells caused by an inappropriate collection of blood samples has to be carefully avoided [199]. Second, low yields of ctDNA recovered after bisulfite conversion could impair the downstream workflow. More in detail, even if bisulfite treatment is the gold standard method for mapping methylated cytosines, it presents some hindrances connected with the wet lab procedure that may cause further loss of ctDNA. Finally, in the perspective of translating the analysis of cfDNA methylation into clinical practice, it is necessary to be aware that changes in cfDNA methylation have been observed not only in cancer but also in other situations, including lupus erythematosus [200], liver fibrosis [201], and diabetes [202]. This could be a challenge, especially in the context of cancer screening.

Moreover, from an economical point of view, the new high-throughput technologies (i.e., microarrays and NGS) applied to the DNA methylation analyses present some limits connected with their relatively elevated costs for use in the clinical routine. Nevertheless, it has to be taken into consideration that the accuracy of the information obtained from these approaches helps in identifying the appropriate (target) treatment, thereby limiting the high costs incurred for ineffective therapies. Finally, the training of laboratory professionals able to interpret the large amount of data derived from these new platforms will be mandatory to provide a rapid flow of reliable information from bench to bedside, capable of driving clinical decisions in real-time.

Overall, the still increasing knowledge and continuous technological development, when accompanied by careful preparation of personnel in the field of DNA methylation, will lead to further achievements, hopefully, spendable in the clinic in the near future.

## 6. Conclusions

The evaluation of epigenetic biomarkers through a liquid biopsy approach is an emerging field that may hold great potential not only for the screening, diagnosis, and identification of tumor types but also for the prediction of response to therapy and/or progression [196,203]. The benefits of this approach include the ability to evaluate tumor markers using non-invasive methods and to gather information on the status and evolution of the systemic, heterogeneous disease. Nowadays, the evaluation of actionable mutations in liquid biopsy has demonstrated clinical value. Nevertheless, the use of epigenetic alterations as biomarkers is still under-exploited, despite their promising potential. DNA methylation tracking is a powerful method for identifying clinically relevant circulating epigenetic biomarkers, facilitating early cancer detection, and resulting in a substantial reduction in cancer—related mortality [203]. From this perspective, all the different components of the circulating compartment deserve to be thoroughly investigated due to the complementary information they may provide. Even though significant efforts have already been made to develop novel biomarkers, much more progress is required. Additional research is required to determine which methylated markers are most accurate to address the questions that need answering. Large-scale targeted methylation sequencing of cfDNA has demonstrated a high potential both for early cancer diagnosis [199] and, most interestingly, for the correlation of methylation scores with treatment outcomes [203]. Since extracellular vesicles carry a molecular “fingerprint” of the cell of origin, they could deliver precious information concerning the cancer status, being prospective biomarkers for melanoma diagnosis or prognosis [145,204]. Finally, circulating melanoma cells, which remain largely unexplored from an epigenetic point of view, are still promising in light of the excellent results obtained in other pathologies.

In conclusion, a better understanding of melanoma epigenetics will enable the identification of more specific diagnostic, prognostic, and predictive biomarkers, which will be advantageous in the era of “precision medicine.” Additionally, it is hoped that more potent epigenetic inhibitors can be developed and tested for the treatment of specific melanoma subtypes.

## Figures and Tables

**Table 1:** Hypermethylated genes in melanoma.

Gene Symbol	Gene Name	Relevance to Melanoma	Ref.
*APC*	Adenomatous Polyposis Coli	Decreased expression increases the proliferation potential of melanoma cells. Found in brain metastases in melanoma.	[59,60]
*CDH11*	Cadherin 11	The hypermethylated *CDH11* facilitates the scattering of tumor cells by loosening contacts between them. Increased proliferation following its inactivation may encourage further establishment at secondary sites, contributing to melanoma progression.	[61,62,63]
*CDH13*	Cadherin 13	The loss of CDH13 is involved in the development of malignant melanoma. Found in brain metastases in melanoma.	[55,59,64]
*CDKN2A*	Cyclin-Dependent Kinase Inhibitor 2A	The hypermethylated *p16^INK4A^* promoter has been predominantly observed in NRAS-mutated metastatic melanomas.	[41,55,65,66,67,68,69,70,71,72,73]
*CLDN11*	Claudin 11	Its methylation level is a potential tool to help discriminate between malignant melanoma and nevus cell nevi.	[74,75]
*DAPK*	Death-Associated Protein Kinase	Detected in patients with both cutaneous and uveal melanoma: its epigenetic silencing is a common mechanism for tumor formation.	[65,76,77]
*ESR1*	Estrogen Receptor 1	The detection of methylated *ESR1* in tissues or sera correlates with tumor progression and is, therefore, of prognostic importance in melanoma patients. In addition, it may identify a population of patients with poor response to systemic therapy, for whom alternative treatment management should be considered.	[55,59,78]
*FES*	FES Proto-Oncogene, Tyrosine Kinase	Its downregulation correlates with poor OS. FES loss drives tumor progression of BRAF V600E-induced murine melanoma.	[79]
*MAPK13*	Mitogen-Activated Protein Kinase 13	Its epigenetic silencing contributes to melanoma progression: restoration of its expression in melanoma cells with *MAPK13* promoter methylation reduces these cells’ proliferative capacity.	[63,75]
*MEOX2*	Mesenchyme Homeobox 2	This gene’s degree of DNA methylation can predict the prognosis of melanoma patients. Its methylation is associated with melanoma progression and/or poor survival.	[80,81]
*MGMT*	O6-Methylguanine-DNA Methyltransferase	Its epigenetic silencing was associated with a better response to DTIC/TMZ therapy and longer PFS in patients with stage IV melanoma and patients with stage III melanoma treated with melphalan locoregional chemotherapy.	[65,72,76,78,82,83,84,85]
*MITF*	Melanocyte Inducing Transcription Factor	The *MITF* gene body was found to be hypermethylated in primary tumors compared to metastases.	[86,87]
*OLIG3*	Oligodendrocyte Transcription Factor 3	This gene’s degree of DNA methylation can predict the prognosis of melanoma patients. Its methylation is associated with melanoma progression and/or poor survival.	[80,81]
*OVOL1*	Ovo Like Transcriptional Repressor 1	Patients with high OVOL1 expression in the primary tumor had a significantly better prognosis than those with low expression.	[80]
*PD-L1*	Programmed Cell Death 1 Ligand 1	Decreased PD-L1 expression correlates with a shorter patient OS.	[88]
*PON3*	Paraoxonase 3	This gene’s degree of DNA methylation can predict the prognosis of melanoma patients. Its hypermethylation is significantly elevated in patients with metastatic melanoma.	[80,81]
*PTEN*	Phosphatase And Tensin Homolog	Reduced OS and DFS in stage III/IV patients. Found in brain metastases in melanoma.	[55,59,89,90,91,92]
*RARβ2*	Retinoic Acid Receptor Beta 2	Correlated with Breslow thickness of the primary tumor: its silencing may be a key epigenetic factor in melanocyte transformation and progression of the primary lesion. Found in brain metastases in melanoma.	[55,59,76,78,85,93,94]
*RASSF1A*	Ras-Association Domain Family Member 1	Detected in patients with cutaneous and uveal melanoma. It can predict the response of patients with stage IV melanoma to biochemotherapy. Found in brain metastases in melanoma.	[47,49,55,59,65,76,77,78,93,95,96,97]
*SOCS1*/*2*	Suppressor of Cytokine Signaling 1/2	Frequently found hypermethylated in the serum of melanoma patients or melanoma cell lines.	[72,94]

**Table 2:** Hypomethylated genes and sequences in melanoma.

Gene/Sequence Symbol	Gene/Sequence Name	Relevance to Melanoma	Ref.
*DSS1*	Deleted in Split-Hand/Split-Foot 1	Its increased expression has been associated with the presence of metastases, ulceration, and reduced OS and DFS, so that it may be used as a biomarker of poor prognosis in melanoma patients.	[131]
*LINE-1*	Long Interspersed Nuclear Element-1	Hypomethylation increase is correlated with advanced stages and a worse prognosis.	[85,130,132]
*MAGE-1*/*2*/*3*/*4*	Melanoma-Associated Antigen 1/2/3/4	Frequently hypomethylated in melanoma cell lines.	[93,133]
*Maspin*	Maspin	It behaves like a TSG in breast and prostate cancer, but its role in melanoma is controversial.	[85,134,135]
*PDGFD*, *THRB*, *ZEB1*	Platelet-Derived Growth Factor D, Thyroid Hormone Receptor Beta, Zinc Finger E-Box Binding Homeobox 1	Its higher expression in NRAS^Q61^-mutated melanomas has been associated with patients’ survival time. It could be a potential candidate for drug development for NRAS-mutant melanomas.	[136]
*PD-L2*	Programmed Cell Death 1 Ligand 2	Predictor of longer PFS in patients referred for anti-PD-1 immunotherapy.	[137]
*TBC1D16*	TBC1 Domain Family Member 16	Associated with increased clinical response to BRAF inhibitors in patients harboring the *BRAF* V600E missense mutation.	[80,138]
*TKTL1*	Transketolase Like 1	It increases the metastatic potential of melanoma cells by contributing to the enhancement of the ’Warburg effect.”	[139]
